# Population Genetics of *Culex tritaeniorhynchus* (Diptera: Culicidae) in Türkiye

**DOI:** 10.1007/s11686-024-00844-9

**Published:** 2024-04-09

**Authors:** Fatma Bursali, Fatih Mehmet Simsek

**Affiliations:** Faculty of Science, Department of Biology, Aydın Adnan Menderes University, Aydın, 09100 Türkiye

**Keywords:** *Culex tritaeniorhynchus*, Population genetics, mtND5, mtCOI, Distribution, Türkiye

## Abstract

**Purpose:**

Mosquitoes are important vectors of pathogens that can affect humans and animals. *Culex tritaeniorhynchus* is an important vector of arboviruses such as Japanese encephalitis virus, West Nile virus among various human and animal communities. These diseases are of major public health concern and can have huge economic and health burdens in prevalent countries. Although populations of this important mosquito species have been detected in the Mediterranean and Aegean regions of Türkiye; little is known about its population structure. Our study is to examine the population genetics and genetic composition of *Cx. tritaeniorhynchus* mosquitoes collected from several localities using cytochrome oxidase subunit I (COI) and the NADH dehydrogenase subunit 5 genes (ND5). This is the first extensive study of *Cx. tritaeniorhynchus* in the mainland Türkiye with sampling spanning many of provinces.

**Methods:**

In this study, DNA extraction, amplification of mitochondrial COI and ND5 genes and population genetic analyses were performed on ten geographic populations of *Culex tritaeniorhynchus* in the Aegean and Mediterranean region of Türkiye.

**Results:**

Between 2019 and 2020, 96 samples were collected from 10 geographic populations in the Aegean and Mediterranean regions; they were molecularly analyzed and 139 sequences (50 sequence for COI and 89 sequence for ND5) were used to determine the population structure and genetic diversity. For ND5 gene region, the samples produced 24 haplotypes derived from 15 variable sites and for COI gene region, 43 haplotypes were derived from 17 variable sites. The haplotype for both gene regions was higher than nucleotide diversity. Haplotype phylogeny revealed two groups present in all populations. AMOVA test results show that the geographical populations were the same for all gene regions. Results suggest that *Cx. tritaeniorhynchus* is a native population in Türkiye, the species is progressing towards speciation and there is no genetic differentiation between provinces and regions.

**Conclusion:**

This study provides useful information on the molecular identifcation and genetic diversity of *Cx. tritaeniorhynchus;* these results are important to improve mosquito control programs.

## Introduction

*Culex* is an important and diverse genus that encompasses more than 770 species grouped into twenty-six subgenera, several of which are of major public health concern [1]. *Culex tritaeniorhynchus* Giles is a cosmopolitan species widely distributed in Africa, Southeast Asia, Middle East and Europe. It primarily breeds in marshes, paddy fields. Taxonomically, this species is a member of the Vishnui subgroup along with *Cx. pseudovishnui, Cx. vishnui, Cx. perplexus* and *Cx. incognitus* [2, 3]. Its distribution around the world seems to be dependent on both climate and altitude [4]. It has a patchy distribution, restricted in some provinces in the Aegean, Mediterranean, and Thrace regions in Türkiye [5–8]. It is a zoophilic and exophilic species that can acquire zoonotic pathogens from natural and/or amplifying reservoir hosts including cows, birds, pigs, horses etc [9–11].

*Culex tritaeniorhynchus* is an important vector of arboviruses such as Japanese encephalitis virus (JEV), Rift valley fever, West Nile virus (WNV) and Tembusu virus among various human and animal communities [12]. These *Culex*-transmitted disease can leave huge economic and health burdens in prevalent countries. WNV and Japanese encephalitis (JE) are one of the serious transmitted infections. They are caused by RNA viruses in the Flaviviridae family and similarly affect the central nervous system of host. WNV is prevalent in Africa, Europe, the Middle East, North America and West Asia [13, 14]. There have been reports of WNV cases and outbreaks in several European countries with the largest occurring in Israel, Greece, Romania, and Russia; between 2010 and 2022. There has been over 5,800 reported human cases and 378 deaths reported [15, 16].

JE, in contrast, is endemic in rural agricultural areas in the East Asia, with an estimated 70,000 cases and a mortality rate of 25–30% reported annually. Children aged ≤ 12 years are the most affected group [17, 18]. Although no JEV has been reported in Türkiye, there have been outbreaks of WNV; 47 WNV cases with 10 fatalities in 2010 [19]. In the following period, 7, 23 and 10 WNV cases were reported in Türkiye in 2017, 2018 and 2019, respectively [20]. Therefore, effective vector control, early detection of new vector species and ongoing vector surveillance are very important in combating these arboviral diseases. The success of vector control efforts to accurately identify the targeted mosquito species, depends on a correct understanding of their biology and ecology. Additionally, many taxonomists evaluate behavioral and population biology data together in identifying and classifying a species [21].

In recent years, numerous molecular phylogeny and population genetics studies have estimated the patterns of relatedness among and between important mosquito species [22–26]. Molecular markers have been developed to distinguish and identify mosquito species, replacing the limitations of morphological identification, which can be challenging for closely related species and requires well-trained technicians [27]. DNA barcoding markers are frequently used for mosquito species differentiation. These studies are important as they provide valuable information on past biogeographic events and potential associations with life history traits of insect vectors. In Türkiye several studies have looked at the population structure of mosquito species such as *Aedes zammitii* [28], *Ae. phoeniciae* [29], *Ae. albopictus* [30], *Ae. aegypti* [31], *Cx. pipiens* [32]. Most recently, Gunay et al. [7] investigated the taxonomic status of the *Culex* fauna in Türkiye using both morphological and DNA barcoding techniques. This work successfully determined or confirmed the presence of *Cx. tritaeniorhynchus* and 15 other *Culex* species, including four newly discovered species; however, no reports exist on the genetic structure of *Cx. tritaeniorhynchus*. Population genetic studies are important for the development of vector control measures, especially genetic control, to prevent or reduce the effects of epidemic diseases. mtCOI DNA barcode regions are commonly used to distinguish mosquito species [33, 34]. Ergunay et al. [35] explored the prevalence and distribution of WNV in potential vectors (mosquitoes) and diverse animal species across 15 Turkish provinces between 2011 and 2013. Notably, WNV RNA was detected in a limited number of mosquito pools, including *Aedes caspius, Cx pipiens, Cx. quinquefasciatus*, and *Cx. perexiguus*. Interestingly, all identified WNV strains belonged to lineage 1 clade 1a. Other field studies in Anatolia and Thrace regions also found WNV lineage 1 in similar mosquito species and migratory birds [36–38]. These findings suggest widespread WNV activity across the investigated regions of Türkiye.

Mitochondrial DNA (mtDNA) is commonly used in insect ecology research. Its benefits include ease of use and specific features. Several studies worldwide have explored the population genetics of *Cx. tritaeniorhynchus* [10, 12, 39, 40]. These studies have reported high haplotype diversity and low nucleotide diversity in different *Cx. tritaeniorhynchus* populations (mainland China), which are attributed to factors such as rapid population expansion, genetic variation, and bottleneck effects [41, 42]. Additionally, it is considered that *Cx. tritaeniorhynchus* can cover distances (~ 2–7.5 km), so there might be more gene flow among populations [42]. Elevation is considered an important factor affecting the distribution of this species *Cx. tritaeniorhynchus* [12]. A recent study in Cambodia employed COI sequences to investigate the phylogeny and spatial distribution of *Cx. tritaeniorhynchus* [43]. Similarly, a study in Thailand used geometric morphometrics and DNA barcoding on *Culex vishnui* Subgroup, which includes *Cx. tritaeniorhynchus*, to inform effective vector control of JE in the region [44]. While COI remains the gold standard for barcoding and species identification [45, 46], incorporating additional markers like ND5 can provide valuable complementary information about genetic diversity and population structure within a species and can offer insights into evolutionary rates or neutrality tests [47, 48]. This proves particularly beneficial when studying under-investigated regions or species with limited COI data. However, we acknowledge a limitation: the absence of ND5 data from other geographic regions like Cambodia and Thailand [43, 44], hinders direct comparisons with those populations. To address this, we propose that future studies in this region consider including ND5 analysis alongside COI to facilitate broader comparisons and a more comprehensive understanding of the species’ distribution and evolutionary history. To our knowledge, no detailed study has been conducted on the population genetics of the *Cx*. *tritaeniorhynchus* populations in Türkiye. Additionally, no studies have investigated the population genetics of *Cx. tritaeniorhynchus* using the mtND5 gene region.

This study aims to be the first to investigate the population structure and genetic variation of *Cx. tritaeniorhynchus* populations in the Aegean and Mediterranean regions of Türkiye using both mtCOI and mtND5, providing valuable information on the population dynamics of this species.

## Materials and methods

### Specimen Collection

**Fig. 1:**
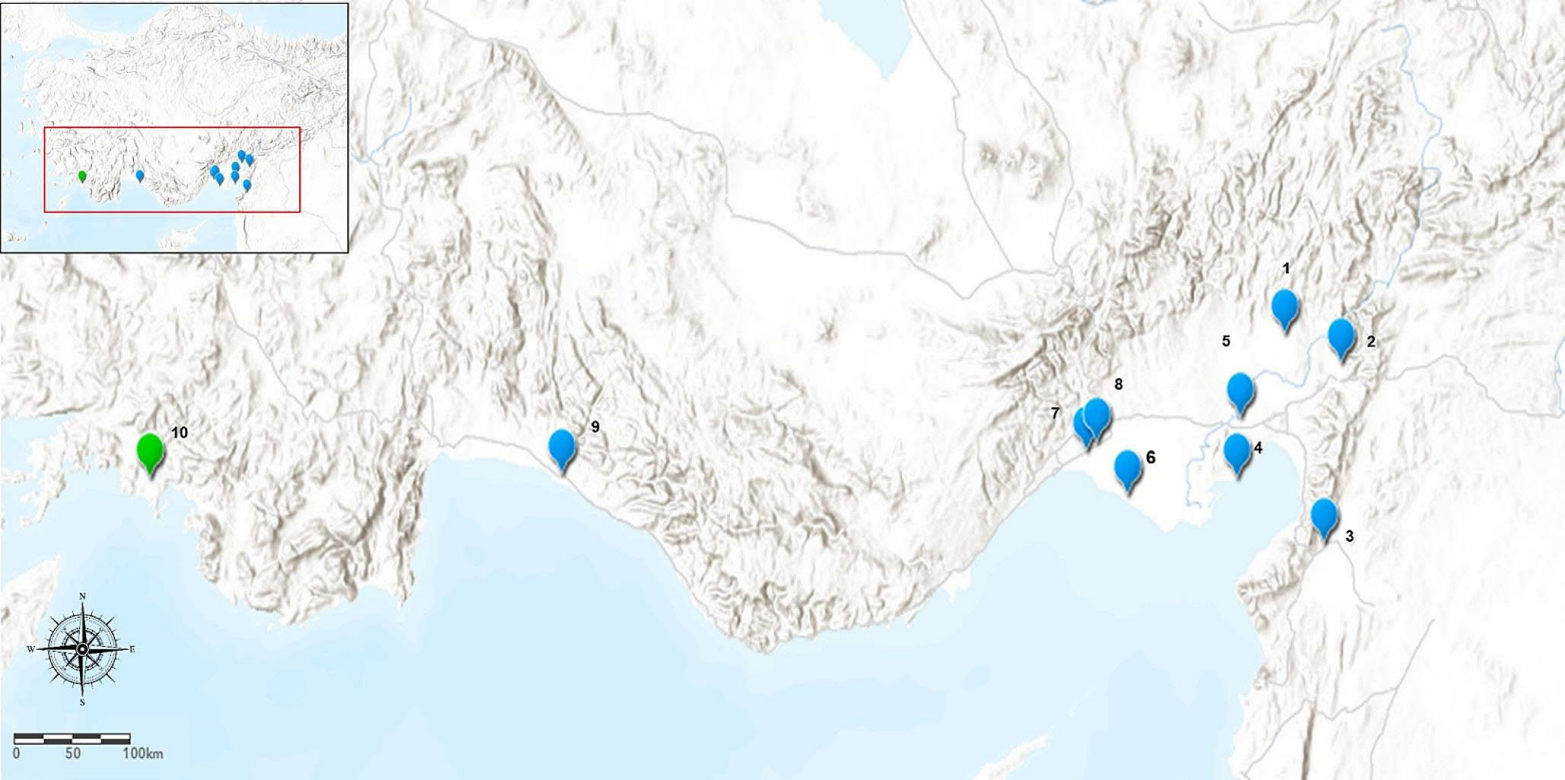
Sampling localities of *Cx. tritaeniorhynchus* populations (1. Osmaniye-Kadirli; 2. Osmaniye-Duzici; 3. Hatay-Kırıkhan; 4. Adana-Yumurtalık; 5. Adana-Ceyhan; 6. Adana-Tuzla 7. Mersin-Tarsus; 8. Mersin-Huzurkent; 9. Antalya-Manavgat 10. Mugla-Dalaman). The map was created using ArcGIS version 10.3. Green symbol represents province in the Aegean region and blue symbols represent provinces in the Mediterranean region

*Cx. tritaeniorhynchus* samples were collected from different districts between the months of May and September, over the 2019–2020 period (Table 1; Fig. 1). Ten geographic populations were selected from all known areas of this species has been detected in. CDC-light traps placed outside barns overnight and mouth aspirators were used to collect adults flying in and around outbuildings.

**Table 1:** Mosquito sampling localities and sample numbers

Region	Province	Locality No	Locality	Latitude	Longitude	Total number of samples collected	Number of samples studied (DNA)
**Mediterranean**	Osmaniye	1	Kadirli	37.347124	36.062823	24	8
		2	Duzici	37.279820	36.453389	24	8
	Hatay	3	Kırıkhan	36.519622	36.375785	24	16
	Adana	4	Yumurtalık	36.768636	35.785260	24	4
		5	Ceyhan	37.034968	35.782780	24	8
		6	Tuzla	36.686331	35.082429	24	4
	Mersin	7	Tarsus	36.908078	34.936978	24	8
		8	Huzurkent	36.883333	34.816667	24	8
	Antalya	9	Manavgat	36.783333	31.433332	24	16
**Aegean**	Mugla	10	Dalaman	36.7131	28.7925	24	16

The adult specimens were transported to the laboratory in paper cups covered with gauze and were used in systematic evaluations under stereomicroscopes and identification key [49]. Identified samples were labelled and stored within 95% ethanol at 20^o^C prior to DNA extraction.

### DNA Extraction, Amplification, and Sequencing

Total DNA extraction of individuals belonging to each population was performed using Invitrogen PureLink genomic DNA isolation kit as instructed by manufacturer. The total number of samples collected, and the number used for molecular studies is given in Table 2. Extracted DNA was stored at − 20°C until PCR amplification. Two mitochondrial gene regions: COI and ND5 were used for molecular studies as described by Folmer et al. [50] and Birungi and Munstermann [51]. The universal LCO1490 and HC02198 barcoding primers were used to amplify the barcode region of the mtCOI gene (658-bp after primer removal), and PCR amplification conditions comprised initial denaturing at 95°C for 5 min, then 34 cycles of 95°C for 30 s, 48°C for 30 min and 72°C for 45 s, followed by a 5-min extension at 72°C and a 4°C hold. The mtND5 gene (approximately 450 bp) was amplified using primers ND5-F and ND5-R. Reactions comprised initial denaturing at 98°C, 2 min, then 30 cycles of 72°C, 45°C, 30 s, 72°C, 45 s, followed by a 5-min extension at 72°C and a 4°C hold. PCR master mix was prepared using Ampliqon Taq 2x master mix according to the manufacturer’s instructions with a final volume of 25 µL each. The PCR reactions contained 12.5 µl PCR mix, 0.25 µl 20 µM each of primers, 1 µl template DNA and 11 µl ddH_2_O. The PCR reactions were performed using a T100 Thermal Cycler (Bio-Rad, Hercules, CA). Subsequently, amplified fragments were loaded on 1% agarose gel, stained with Safeview™ and visualized under UV light. Visualized products were purified by PCR purification kit and the cleaned PCR products sent out to Macrogen, Amsterdam, Netherlands for sequencing.

### Population Genetics, Differentiation and Data Analyses

The ND5 and COI sequences obtained using forward and reverse PCR primers were visualized and edited using the BioEdit ver 7.0.9 software [52] and MEGA7 software [53], respectively. Sequences were purified based on high-quality read peaks for each gene region using MEGA7and aligned using the Clustal W algorithm. To minimize positional dissimilarities between sequences of varying lengths, the alignments were manually adjusted after trimming the start and end points. All missing data and gaps within the sequences were removed. The sequence arrangement was then manually reassessed in an attempt to minimize the positional dissimilarity. All missing data and gaps within the sequences were removed. The sequences were compared within themselves and with the previous DNA sequences obtained from the Genbank, and sequences with more than 97% similarity were used in the study. Multiple sequence alignment of sequences was performed using the Clustal W algorithm in MEGA7 software [53].

Polymorphic sites (S), numbers of haplotypes (N), haplotype diversity (Hd), and nucleotide diversity (π) of *Cx. tritaeniorhynchus* populations were assessed using DnaSP 5.0 software [54] for each gene region. Nucleotide diversity (*π*) and haplotype diversity (Hd) are two major indicators to calculate the diversity of species populations among different geographical strains. Pairwise FSTs values of the genetic differentiation among the populations, selective neutrality tests including Fu’sFS statistics and Tajima’s D tests, and analysis of molecular variance analysis (AMOVA) were performed in Arlequin 3.5 software [55]. Median-joining Network analysis to show the relationship between haplotypes were performed using Network 10.2 software [56]. Also, all p values were corrected using Holm’s correction method. A phylogenetic tree was constructed using the neighbor-joining algorithm in MEGA7 software. *Culex pipiens* mtCOI and mtND5 sequences from GenBank database was used as an outgroup. The analysis was run on 1000 replicates for inferred bootstrap consensus. The best fit model was selected using MODEL TEST 3.0 software [57, 58].

## Results

*Culex tritaeniorhynchus* samples were obtained from 10 geographic populations after sampling mosquitoes in the Aegean and Mediterranean regions. DNA from the mtCOI and mtND5 gene regions from a total of 96 *Cx. tritaeniorhynchus* samples were amplified and after sequence analysis, only 50 mtCOI sequence data and 89 mtND5 sequence data were suitable and used for phylogenetic studies (Haplotypes’ GenBank accession numbers for mtCOI: PP447202- PP447244; for mtND5: PP459986- 460,009).

According to the data from 89 mtND5 (410 bp) nucleotide sequences, the G + C ratio of these sequences was calculated as 0.240 and found lower than the A + T ratio. All sequences generated 24 haplotypes generating from 15 polymorphic sites, and Hd and pi values were determined as 0.841 and 0.01783, respectively. The highest number of haplotypes were determined in the Antalya population (h = 9, Hd = 0.886 and pi = 0.01681), the lowest haplotype number was determined in the Mersin population (h = 6, Hd = 0.889 and pi = 0.01857). Tajima’s D and Fu’s FS tests were conducted for neutrality analysis to detect the history of geographical population size; Fu’s FS tests were positive in the majority of populations (except Antalya population), the values ​​were found to be statistically insignificant. Tajima’s D test results were positive for all populations and this value was statistically significant for Osmaniye, Hatay and Adana populations. The genetic diversity values of the mtND5 gene region are summarized in Table 2.

For mtCOI gene region, 50 (final edited product size 450 bp) nucleotide sequences were used for haplotype analysis. The G + C ratio of these sequences was ranged between 0.240 and 0.315. All sequences generated 43 haplotypes originating from 17 polymorphic sites. Calculated Hd and Pi values were 0,992 and 0,01433, respectively. The highest haplotype number was determined in Antalya (h = 14) similar as in the ND5 gene region, while the lowest haplotype number was determined in Adana (h = 5) population. Fu’s FS tests for neutrality tests were negative in most populations (except Adana and Mugla population), these values ​​were found to be statistically significant (*p* < 0,05). Tajima’s D test results were positive for all populations and this value was statistically significant like mtND5 gene region. Between populations, although the values ​​were positive, they were not significant (*p* > 0,05) and the Tajima’s D value could not be calculated for Adana and Mugla populations. The genetic diversity values ​​of the mtCOI gene region are summarized in Table 2.

**Table 2:** Genetic diversity of *Culex tritaeniorhynchus*, using mtCOI and mtND5 gene sequences

Sequence	Localite	n	S	p	H	Hd	Tajima’s D test	Fu’S FS statistic	G + C content
**ND5**	**Osmaniye**	16	14	0,01715	8	0,883	**2,16375**	0,856	0,240
	**Hatay**	16	15	0,01926	8	0,850	**2,47366**	1,219	0,241
	**Adana**	16	15	0,01884	7	0,792	**2,33645**	2,154	0,240
	**Mersin**	10	15	0,01857	6	0,889	1,57130	1,241	0,241
	**Antalya**	15	14	0,01681	9	0,886	1,96326	-0,359	0,239
	**Mugla**	16	15	0,01762	7	0,800	1,93205	1,919	0,240
	**Total**	89	15	0,01783	24	0,841	**3,66592**	-2,511	0,240
**COI**	**Osmaniye**	8	15	0,01525	8	1,000	0,68611	**-2,849**	0,315
	**Hatay**	12	16	0,01307	12	1,000	0,56599	**-6,922**	0,314
	**Adana**	2	5	0,01027	2	1,000		1,609	0,320
	**Mersin**	11	15	0,01437	10	0,982	1,25437	**-3,303**	0,315
	**Antalya**	14	16	0,01424	14	1,000	1,22568	**-8,570**	0,314
	**Mugla**	3	7	0,00958	3	1,000		0,308	0,314
	**Total**	50	17	0,01433	43	0,992	**2,04463**	-41,529	0,315

N = Number of samples; S = Number of variable sites; H = Number of haplotypes; Hd = Haplotypes diversity; p = Nucleotide diversity

### Genetic Relationships among Geographical Populations in Türkiye

Genetic relationships among 6 geographical populations were determined using pairwise *Fst* distance. *Fst* distance values ranged between − 0,06268 and 0,06835 for ND5 gene region. The *Fst* values for all population pairs were detected as insignificant for mtND5 gene region (Table 3).

**Table 3:** Pairwise *Fst*’s of 6 populations using partial sequences of mtND5. Statistically significant values were marked in bold

	Osmaniye	Hatay	Adana	Mersin	Antalya	Mugla
**Osmaniye**						
**Hatay**	-0,02324					
**Adana**	-0,02574	-0,05761				
**Mersin**	0,06835	-0,02439	-0,01284			
**Antalya**	0,05185	-0,02155	-0,01545	-0,05282		
**Mugla**	-0,06268	-0,03079	-0,03817	0,05185	0,03372	

*Fst* distance values ranged between − 01980 and 0.29660 for mt mtCOI gene region. While between Osmaniye-Mugla, Hatay-Adana, Adana–Antalya population pairs, significant differences were determined between the populations, the differences between other populations were found to be insignificant (*p* > 0.05) (Table 4).

**Table 4:** Pairwise *Fst*’s of 6 populations using partial sequences of mtCOI. Statistically significant values were marked in bold

	Osmaniye	Hatay	Adana	Mersin	Antalya	Mugla
**Osmaniye**	0.00000					
**Hatay**	0.04298	0.00000				
**Adana**	0.03696	**0.29660**	0.00000			
**Mersin**	0.00445	0.04278	0.10811	0.00000		
**Antalya**	0.04585	0.00568	**0.12220**	-0.01980	0.00000	
**Mugla**	**0.15142**	0.00557	0.49609	0.00633	0.09454	0.00000

### Phylogenetic Analysis of *Culex tritaeniorhynchus* Haplotypes

**Fig. 2:**
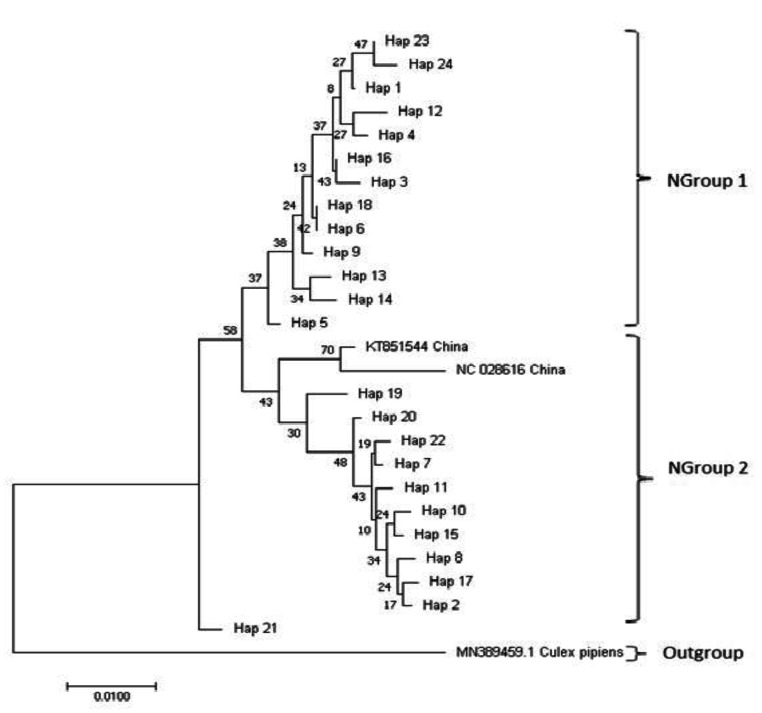
Neighbor-joining tree of *Culex tritaeniorhynchus* haplotypes, Genbank samples and outgroup using T92 + G + I distance model for mtND5 gene region. Bootstrap values were shown under the respective nodes

For the mtND5 gene region, *Cx. tritaeniorhynchus* samples from this study, two samples from China (GenBank number: KT851544, NC_028616) and a *Culex pipiens* (GenBank number: MN389459) as an outgroup were obtained from the GenBank and used in obtaining a phylogenetic Neighbor-joining (NJ) tree using the T92 + G + I method. NJ tree revealed two main groups. The first group (Expressed as NGroup1 on the Fig. 2) was clustered with Hap 1, Hap 3, Hap 4, Hap 6, Hap 9, Hap 12, Hap 13, Hap 14, Hap 16, Hap 18, Hap 23, and Hap 24. The second group (Expressed as NGroup2 on the Figure) was clustered with Hap 2, Hap 5, Hap 7, Hap 8, Hap 10, Hap 11, Hap 15, Hap 17, Hap 19, Hap 20, Hap 22. Hap 21 was in middle of the two groups. The haplotype can be hybrid of the two groups.

**Fig. 3:**
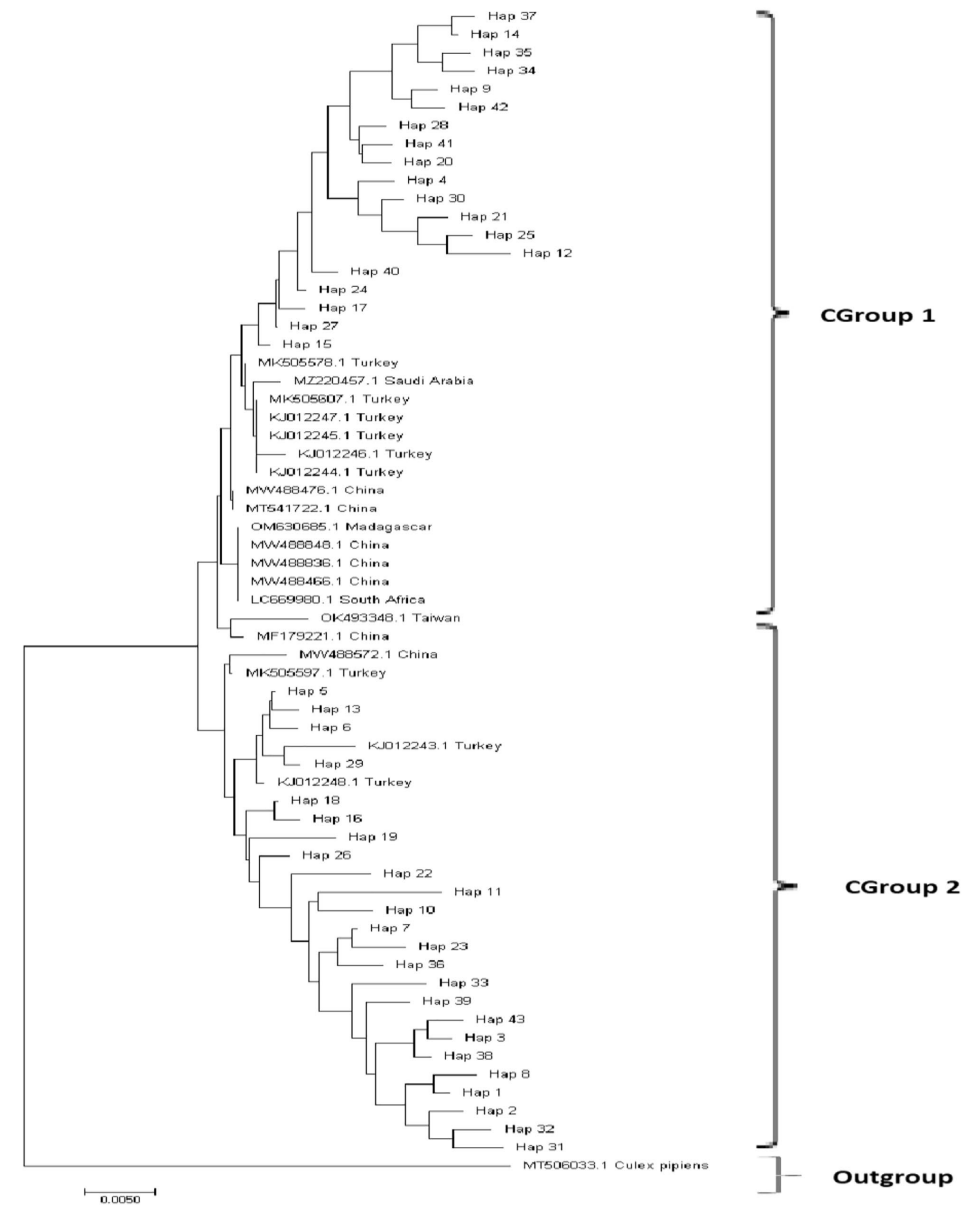
Neighbor-joining tree of *Culex tritaeniorhynchus* haplotypes, Genbank samples and outgroup using T92 + G + I distance model for mtCOI gene region

For the mtCOI gene region, some of the haplotypes obtained showed high similarity with the GenBank samples. However, none of GenBank samples idented 100% with our samples. Phylogenetic tree was constructed using the samples with high blasted GenBank samples and haplotypes, 2 main groups emerged similar to the mtND5 gene region (Fig. 3). Moreover, GenBank samples recorded from Türkiye were also represented in these two groups. All the samples outside of Türkiye were in Cgroup 1.

**Fig. 4:**
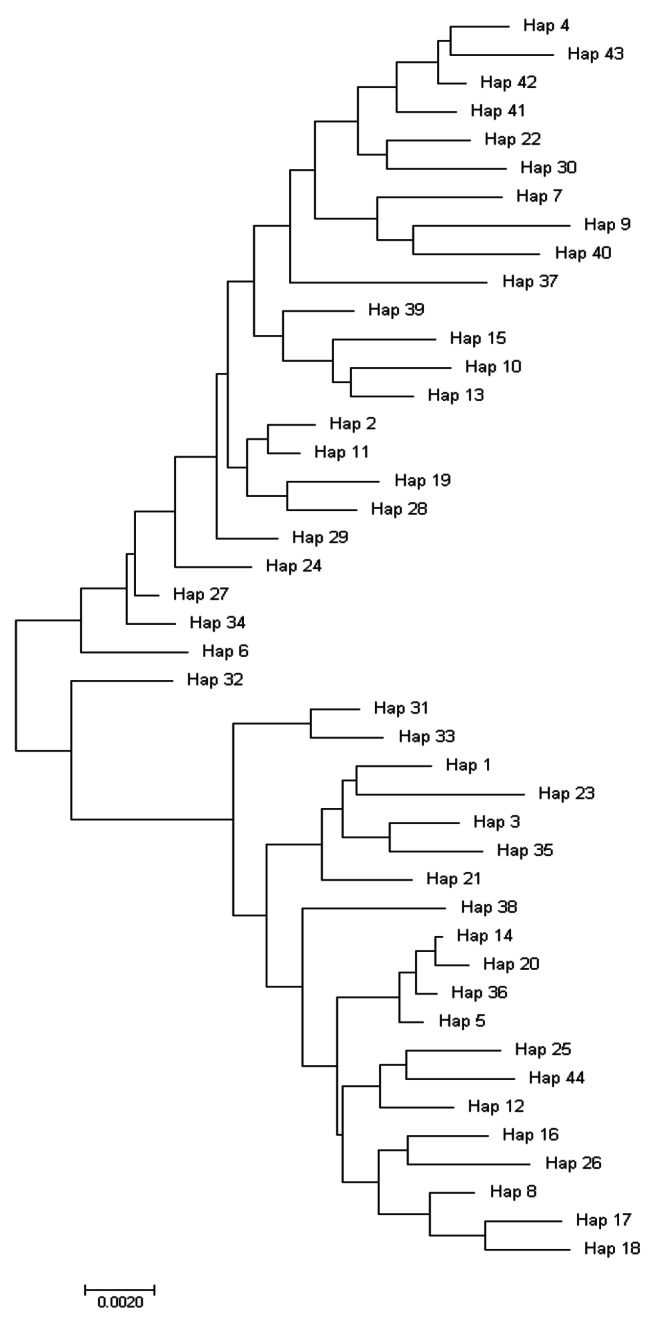
Neighbor-joining tree of *Culex tritaeniorhynchus* concatenated haplotypes

Also, a phylogenetic tree was constructed using concatenated haplotype from each population. The sequences concatenated in Sequence Matrix 1.7 software. The compatibility of the sequences was assessed with using a partition homogeneity test in PAUP 4.0 software (Fig. 4).

### Haplotype Network Analyses

**Fig. 5:**
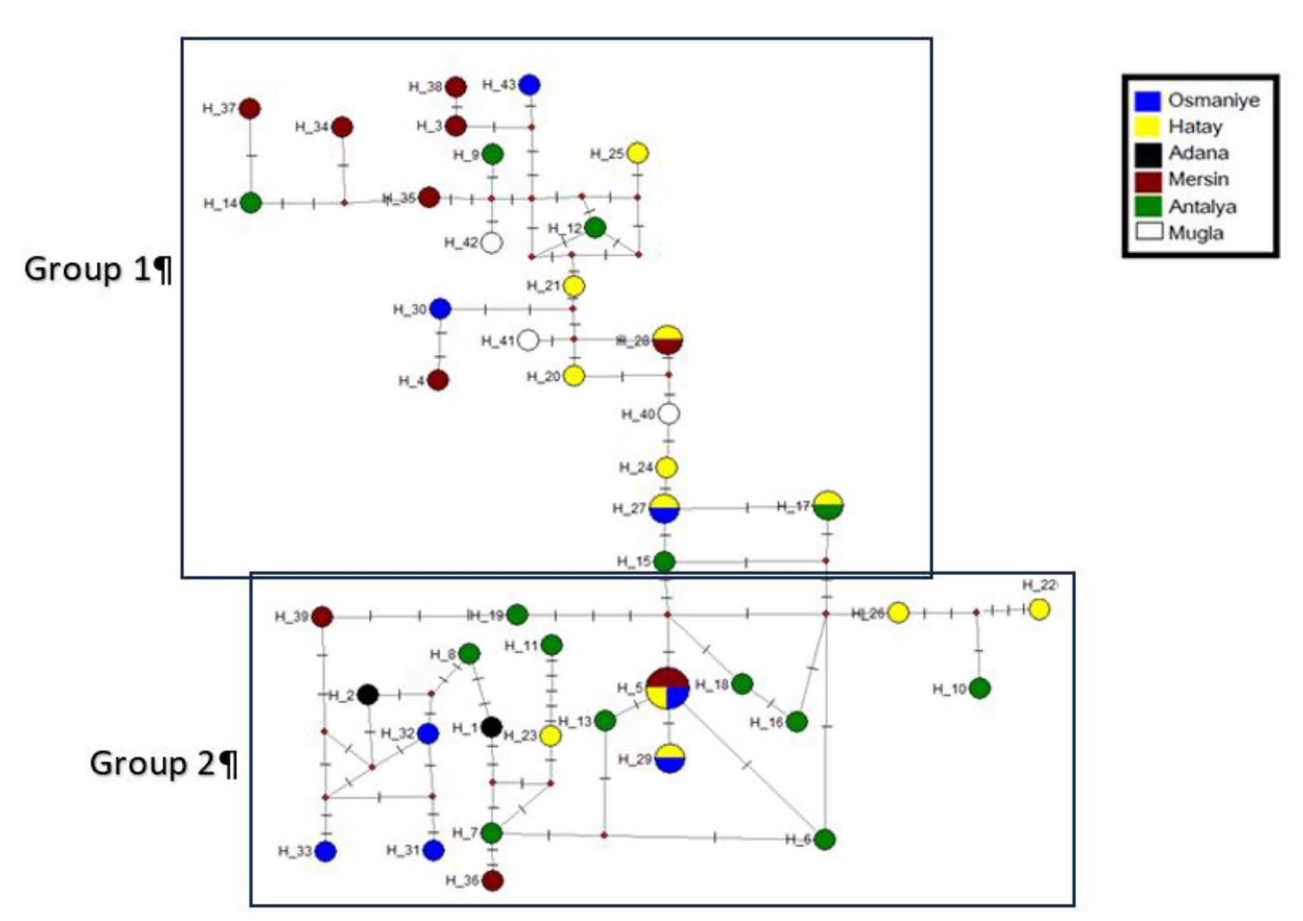
Network of the mtCOI haplotypes of *Culex tritaeniorhynchus*

The relationship between haplotypes were analyzed using the median-joining method in Network 10.2 software. In the network, each circle represents one haplotype, and the circle size is related to the frequency of occurrence of the haplotype. The haplotype network was made with 43 haplotypes for the mtCOI gene region which were clustered into 2 main groups, supporting the NJ tree of mtND5 gene region. Osmaniye, Hatay, Antalya and Mugla populations, included in CGroup 1, was represented by all populations except Adana population. CGroup2 comprised of all populations. Hap 17, Hap 20, Hap 27 was represented higher than other haplotype in CGroup 1 for CGroup 3, the main haplotype was determined as Hap5, and Hap27, Hap17, Hap29 were the other highest frequency haplotypes (Fig. 5).

**Fig. 6:**
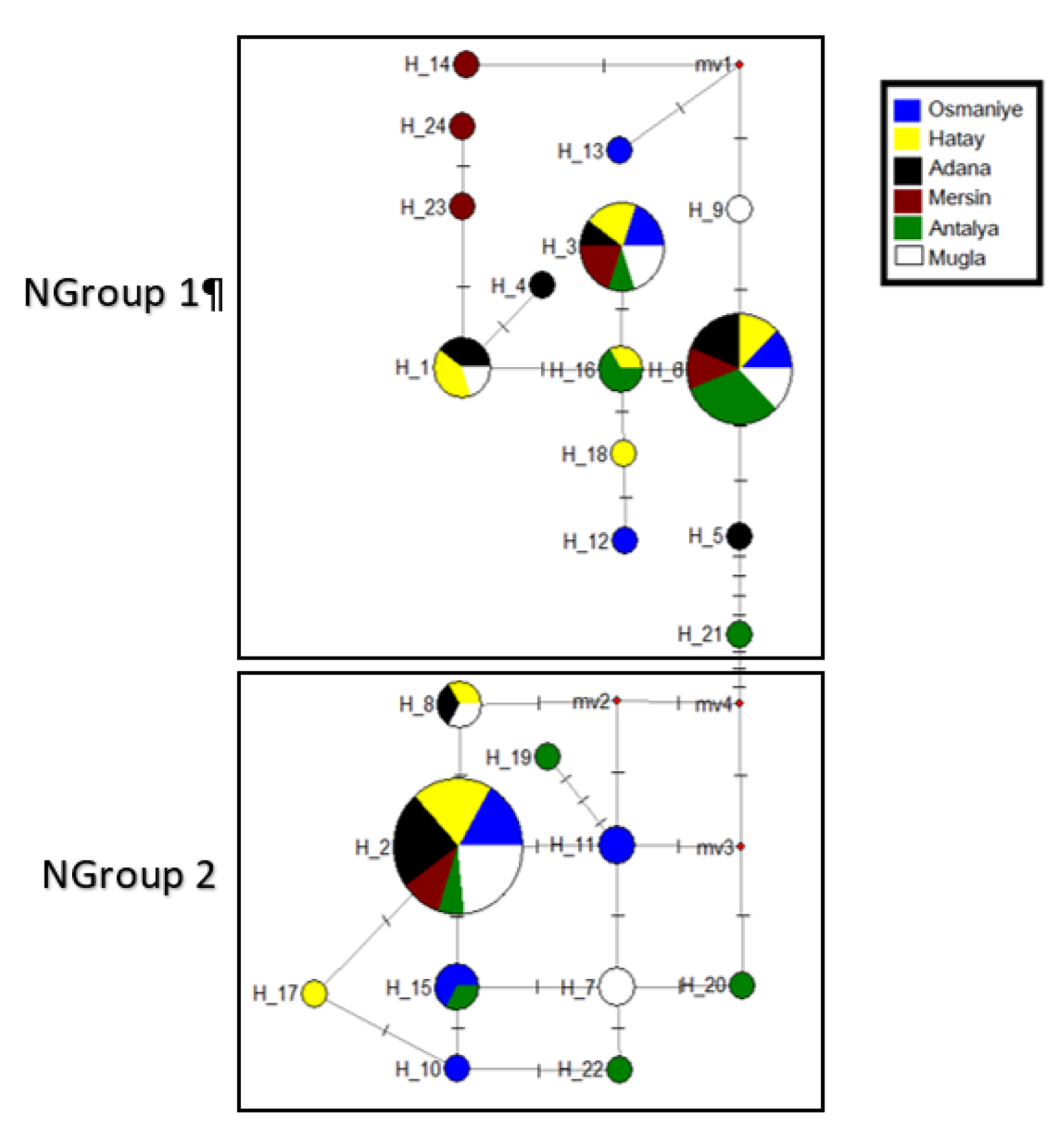
Network of the mtND5 haplotypes of *Culex tritaeniorhynchus*

According to the mtND5 gene region, haplotype network was supported NJ tree results and the haplotypes was clustered on two main groups (NGroup 1 and NGroup 2). NGroup 1 and NGroup 2 were represented by all populations. In NGgroup 1, Hap 6 was determined as the main haplotype, and Hap 3 and Hap 1 was detected other high frequency haplotypes. For NGroup 2, the main haplotype was determined as Hap 2 (Fig. 6).

### Analysis of Molecular Variance (AMOVA) Analysis

We performed the AMOVA test to determine whether there is a difference between the Aegean and Mediterranean populations of Türkiye. For mtCOI gene region, variance component proportion of within populations is high (92.41%), while among geographical strains (Aegean and Mediterranean) and within groups proportion is low (4.05% and 3.54% respectively). F-statistics, among groups, among populations within groups, and within populations were Fsc = 0.03688, Fst = 0.07593, and Fct = 0.04055, respectively, and were found statistically insignificant (Table 5).

AMOVA analysis for the mtND5 gene region gave similar results to the mtCOI gene region; variance component proportion of within populations is high (101.38%), while among geographical strains (Aegean and Mediterranean) and within group proportion is low (-0.20% and − 1.18% respectively). F-statistics, among groups, among populations within groups, and within populations were Fsc = 0.01174, Fst = 0.01378, and Fst = 0.00202, respectively, and were found statistically insignificant (Table 5).

**Table 5:** AMOVA analysis results

	COI				ND5			
Source of variation	d.f.	Sum of squares	Variance components (% variation)	Fixation index	d.f.	Sum of squares	Variance components (% variation)	Fixation index
Among groups	1	4.654	0.14782 (4.05)	Fsc: 0.03688	1	2637	-0.00689 (-0,20)	Fsc: -0.01174
Among populations within groups	4	18.088	0.12902 (3.54)	Fst: 0.07593	4	11,479	-0,04003 (-1,18)	Fst: -0.01378
Within populations	44	148.238	3.36905 (92.41)	Fct: 0.04055	83	286,379	3.45035 (101.38)	Fct: -0.00202
Total	49	170.980	3.64590		88	300.494	3.40344	

## Discussion

Molecular phylogeny is used to study various aspects of evolution, including gene duplication rates, diversification patterns, genetic variation, recombination events, population dynamics, and organismal relationships. This approach often involves combining molecular data with other sources of information [21]. There have been relatively few comparative studies on *Cx. tritaeniorhynchus* population diversity, or variation in vectorial capacity, with the majority characterizing *Cx. tritaeniorhynchus* populations within Asia [40, 41, 59–62]. Historically this is where this species has had the greatest occurrence, abundance, and caused the greatest impact on human health as the major vector of JEV [63]. Studies on this species are limited in Türkiye. The mtCOI gene has been the most frequently used for previous studies of *Cx. tritaeniorhynchus* [40, 41, 59–61, 64] given it contains areas of highly conserved sequence, in combination with sufficiently diverse areas, allowing species discrimination and investigation of maternal inheritance patterns [22, 64, 65]. As a first, our study examined the population genetics and genetic composition of Turkish *Cx. tritaeniorhynchus* mosquitoes collected from all known areas this species has been detected in using mtCOI and mtND5 sequences.

Results on nucleotide diversity and haplotype diversity, two major indicators to calculate the diversity of species populations among different geographical strains, showed that there was a high genetic variation detected in *Cx. tritaeniorhynchus* populations in Türkiye. Similarly, Rajavel et al. [61] and Airi et al. [66] in India, Jeffries et al. [67] in Asian, African and Middle Eastern populations determined high haplotype diversity (~ 0,9−1); while relatively low haplotype diversity (between ~ 0.5–0.8) was determined in the European continent where the species is newly entered. In another study done by Jeffries et al. [67], our results are not compatible with them, the reason for this situation may be due to the fact that not enough samples were used in the study (only 13 samples).

Previous studies have identified 28 [64], 303 [40] and 444 haplotypes [67]. Analysis of regional population groups identified haplotypes in Asia (n = 909, Hd = 0.97, Pi = 0.02203), 4 in Australia (n = 19, Hd = 0.73, Pi = 0.00565), 19 in Africa (n = 34, Hd = 0.96, Pi = 0.00819), 4 in the Middle East (n = 4, Hd = 1.00, Pi = 0.01087), 8 in Eurasia (n = 22, Hd = 0.86, Pi = 0.00794) and 4 in Europe (n = 19, Hd = 0.64, Pi = 0.00184). The only previous study for European *Cx. tritaeniorhynchus* found two haplotypes within the same population, collected in a single rice field in western Greece [11]. Neutrality tests for the mtND5 gene region also supported this situation and Tajima’s D test values ​​were determined as positive for all populations and these values ​​were statistically significant for Osmaniye, Hatay and Adana populations [68, 69]. Fu’s FS statistical values were similarly positive except for the Antalya population. The Antalya population may indicate a re-expansion, possibly due to frequent control studies against mosquito species, because both the mtCOI and mtND5 gene regions gave negative and significant Fu’s FS statistical values. On the other hand, Tajima’s D values for the mtCOI gene region were positive, as in the mtND5 gene region, but all of the values ​​were statistically insignificant. Fu’s FS statistical values was negative in all populations (except Mugla and Adana populations) in other populations, and these values ​​were statistically significant. This is evidence of an excess of low-frequency haplotypes as expected from population growth or may result from secondary contact between prior allopatric populations [70]. The different results observed in Tajima’s D and Fu’s FS statistics may possibly be due to the previous observation of variation in the mtCOI gene and, accordingly, the insufficient number of samples (Table 2). Both NJ trees and Haplotype network trees formed two different groups and all groups were represented by all populations. This situation may have been caused by allopatric speciation and could be shown to cause negative Fu’ FS values ​​for the mtCOI gene region. However, neutrality tests obtained from the mtND5 gene region did not support this situation.

In general, high variation was detected in all populations of the species. On the other hand, high variation is frequently seen in native species [32]. In other studies, high variation was determined in the regions where it is native [40, 41, 67]. While high gene flux was determined in the populations for the mtND5 gene region among the populations, significant differences were determined between the Osmaniye, Mugla and Adana Antalya pairs for the mtCOI gene region. In fact, this is quite logical given the geographical distances. On the other hand, it is not possible to explain the significant difference between Hatay-Adana couples with geographical distance. The reason for this situation may be the geographical barrier created by Taurus and Amanos mountains. In the studies, it has been determined that the mtCOI gene region is more variant and mores ensitive than the mtND5 gene region [30]. Phylogenetic tree and haplotype networks formed two main groups for mtND5 and a third intermediate group was determined between these two main groups. The fact that these groups are represented by all populations is probably due to gene flux between populations of the species, both actively and passively, which causes gene flux between populations. Almost the same situation has been identified for the mtCOI gene region by Xie et al. [40] in Chinese populations. This grouping may be geared towards the speciation of the species. These results were similar in the mtCOI gene region line [7] studied with samples collected from nearby geographical regions (Doğankent Adana), and it was stated in their studies that this situation was related to speciation. This grouping may be important in terms of contributing to the vector competence of the species [7]. Seeing these variations within the species necessitates research on the basis of subspecies. The effect of geographical regions that may cause this situation was investigated with the AMOVA test. However, no significant differences could be determined between the groups for both the mtCOI and mtND5 gene regions.

There have been numerous recent reports of *Cx. tritaeniorhynchus* in countries where it had previously not been reported [71–74] highlighting a trend towards expansion of its known geographical range. Determining the phylogenetic origins of maternal lineages of *Cx. tritaeniorhynchus* can provide some insight into possible movement patterns when compared across countries and regions. During normal daily activity, *Cx. tritaeniorhynchus* are estimated to have an average flight distance of just under 70 m, however, some studies have found that during long-distance wind-assisted dispersal, they are estimated to migrate between 200 and 500 km [75]. The adults over winter and it is thought this species may use a combination of long-distance migration and hibernation in situ, as strategies to survive unfavorable conditions in temperate regions [76]. The ability to disperse over such long distances and adapt to variable conditions is likely to provide more opportunities for range expansion and to increase gene exchange among different populations [40].

Despite the presence of *Cx. tritaeniorhynchus* first being reported in Europe– specifically Albania– in 1960 [77], further published European occurrence reports were scarce until the 2000s, with the species recorded in Greece; including from coastal marsh in Marathon near Athens [72], rice fields in Messolonghi, western Greece [11] and an urban area in Epirus, northwestern Greece [78]. Recent extensive entomological surveys carried out in Albania have identified the presence of *Cx. tritaeniorhynchus* within multiple areas across the country. Concurrently, there has been an increasing trend of incursion, outbreaks and circulation of mosquito-borne arboviruses such as WNV in Europe, with many becoming established and endemic in multiple countries [15, 79].

Our study is the first to assess the distribution pattern and perform molecular screening of *Cx. tritaeniorhynchus* populations in the Aegean and Mediterranean regions of Türkiye. In this study, distribution patterns of *Cx. tritaeniorhynchus* were identified, and genetic characterization of the mosquitoes is reported for the first time in Türkiye. The study targeted the Aegean and Mediterranean regions due to their contrasting geographies: the Aegean’s gentle landscape (plains, hills, and valleys) versus the Mediterranean’s rugged terrain dominated by the Taurus and Amanos Mountains. While both regions share a Mediterranean climate, the Aegean is cooler and wetter. This geographical and environmental variations can influence mosquito breeding and distribution. Also the Taurus Mountains might act as a barrier, promoting genetic differentiation between *Cx. tritaeniorhynchus* mosquito populations in these regions. These findings will enhance our understanding of vector–pathogen dynamics in Türkiye and inform researchers about the possibility of the occurrence of a possible outbreak. It may also facilitate control efforts in many aspects. Türkiye is the bridge between Asia and Europe or Middle East to Eastern part of Europe and many refugees originating from arboviral disease endemic areas use this route to reach continental Europe. The presence and spread of *Cx. tritaeniorhynchus* in Türkiye is an important public health threat not only for the introduction of emerging arboviruses but also for autochthonous West Nile virus circulation. In these aspects, the introduction of national entomological surveillance programs and applying efficient vector control measures are critical factors in reducing the risk of future autochthonous arboviral disease.

## Conclusions

To our knowledge this is the first published study about genetic diversity of *Cx. tritaeniorhynchus* populations in Türkiye. Availability of a broader range of genetic data, with wide coverage of informative genes will be valuable in further understanding the phylogeography, divergence, range expansions and evolution of this species. Also, *Cx. tritaeniorhynchus* populations will greatly expand the utility for comparison and potential for understanding this mosquito species and its contribution to vector-borne disease transmission.

This study also adds valuable information about this mosquito species acting as a significant vector for Japanese encephalitis in various Asian continents. The generated mtCOI sequences could be used as reference nucleotide sequences of the respective haplotypes in future mosquito identification studies and will facilitate the conspecific comparison to reveal the appropriate reason for high intra-specific divergence. Furthermore, different climatic conditions and geographical barriers can be responsible for the genetic variations in as pecies. This information is further used for the effective implementation of region-specific vector control strategies.

## References

[1] Harbach RE (2016) Mosquito Taxonomic Inventory. http://mosquito-taxon omic-inventory.info

[2] Harbach RE (2011). Classification within the cosmopolitan genus Culex (Diptera: Culicidae): the foundation for molecular systematics and phylogenetic research. Acta Trop.

[3] Liu LY, Deng YP, Zhang Y, Wu Y, Fu YT, Liu GH, Liu JH (2023). Characterization of the complete mitochondrial genome of Culex vishnui (Diptera: Culicidae), one of the major vectors of Japanese encephalitis virus. Parasitol Res.

[4] Miller RH, Masuoka P, Klein TA, Kim HC, Somer T, Grieco J (2012). Ecological niche modeling to estimate the distribution of Japanese encephalitis virus in Asia. PLoS Negl Trop Dis.

[5] Alten B, Çaglar SS, Özer N (2000). Malaria and its vectors in Turkey. Eur Mosq Bull.

[6] Ramsdale CD, Snow K (2001). Distribution of the genera Coquillettidia, Orthopodomyia and Uranotaenia in Europe. Eur Mosq Bull.

[7] Gunay F, Alten B, Simsek F, Aldemir A, Linton YM (2015). Barcoding Turkish Culex mosquitoes to facilitate arbovirus vector incrimination studies reveals hidden diversity and new potential vectors. Acta Trop.

[8] Touray M, Bakirci S, Ulug D, Gulsen SH, Cimen H, Yavasoglu SI, Hazir S (2023) Arthropod vectors of disease agents: their role in public and veterinary health in Turkiye and their control measures. Acta Trop 106893. 10.1016/j.actatropica.2023.106893 10.1016/j.actatropica.2023.106893 37004805

[9] Jupp PG, Kemp A, Grobbelaar A, Leman P, Burt FJ, Alahmed M (2002). The epidemic of Rift Valley fever in Saudi Arabia: Mosquito vector studies. Med Vet Entomol.

[10] Sallam MF, Al AAM, Abdel-Dayem MS, Abdullah MAR, Edward PR (2013). Ecological niche modeling and land cover risk areas for rift valley fever vector, Culex Tritaeniorhynchus Giles in Jazan, Saudi Arabia. PLoS ONE.

[11] Lytra I, Emmanouel N (2014). Study of Culex Tritaeniorhynchus and species composition of mosquitoes in a rice field in Greece. Acta Trop.

[12] Liu B, Gao X, Ma J, Jiao Z, Xiao J, Wang H (2018). Influence of host and environmental factors on the distribution of the Japanese encephalitis vector Culex Tritaeniorhynchus in China. IJERPH.

[13] Chancey C, Grinev A, Volkova E, Rios M (2015) The global ecology and epidemiology of West Nile virus. 10.1155/2015/376230. BioMed research international 10.1155/2015/376230 PMC4383390 25866777

[14] Bakonyi T, Haussig JM (2020). West Nile virus keeps on moving up in Europe. Euro surveillance: bulletin Europeen sur les maladies transmissibles = European. Commun Disease Bull.

[15] European Centre for Disease Prevention and Control Weekly updates: 2022 WestNile virus transmission season [Internet]. [Available https://www.ecdc.europa.eu/en/west-nile-fever/surveillance-and-disease-data/disease-data-ecdc, Accessed 24/07/2023]

[16] Campbell Grant L, Hills SL, Fischer, Marc J, Julie A, Hoke Charles H (‎2011)‎ estimated global incidence of Japanese encephalitis: a systematic review. Bull World Health Organ 89(‎10)‎:66–774E. 10.2471/BLT.10.085233 10.2471/BLT.10.085233 PMC3209971 22084515

[17] World Health Organization (2015). Japanese encephalitis vaccines: WHO position paper-February 2015. Wkly Epidemiol Rec.

[18] Kalaycioglu H, Korukluoglu G, Ozkul A, Oncul O, Tosun S, Karabay O, Gozalan A, Uyar Y, Caglayık DY, Atasoylu G, Altas AB, Yolbakan S, Ozden TN, Bayrakdar F, Sezak N, Pelıtlı TS, Kurtcebe ZO, Aydın E, Ertek M (2012). Emergence of West Nile virus infections in humans in Turkey, 2010 to 2011. Euro Surveillance: Bull Europeen sur les maladies Transmissibles.

[19] European Centre for Disease Prevention and Control (ECDC) (2020) Communicable Disease Threats report. 11–17 October 2020, Week 42. ECDC, Stockholm. https://www.ecdc.europa.eu/en/publications-data/communicable-disease-threats-report-11-17-october-2020-week-42

[20] Panda D, Barik TM (2022). Molecular characterization and genetic divergence of seven Culex mosquito (Diptera: Culicidae) species using mt COI gene from Odisha State, India. J Basic Appl Zool.

[21] Cywinska A, Hunter FF, Hebert PD (2006). Identifying Canadian mosquito species through DNA barcodes. Med Vet Entomol.

[22] Fotakis EA, Chaskopoulou A, Grigoraki L, Tsiamantas A, Kounadi S, Georgiou L, Vontas (2017). Analysis of population structure and insecticide resistance in mosquitoes of the genus Culex, Anopheles and Aedes from different environments of Greece with a history of mosquito borne disease transmission. Acta Trop.

[23] Elnour MAB, Gloria-Soria A, Azrag RS, Alkhaibari AM, Powell JR, Salim B (2022). Population genetic analysis of Aedes aegypti mosquitoes from Sudan revealed recent independent colonization events by the two subspecies. Front Genet.

[24] Amorim JA, de Oliveira TMP, de Sá ILR, da Silva TP, Sallum MAM (2023). DNA barcodes of Mansonia (Mansonia) Blanchard, 1901 (Diptera, Culicidae). Genes.

[25] Futami K, Valderrama A, Baldi M, Minakawa N, Rodríguez Marín, Chaves R (2015). New and common haplotypes shape genetic diversity in Asian tiger mosquito populations from Costa Rica and Panamá. J Econ Entomol.

[26] Mewara A, Sharma M, Kaura T, Zaman K, Yadav R, Sehgal R (2018). Rapid identification of medically important mosquitoes bymatrix-assisted laser desorption/ionization time-of-flight mass spectrometry. Parasites Vectors.

[27] Yavasoglu SI, Simsek FM, Ulger C (2016). Distribution pattern and genetic structure of Aedes zammitii (Diptera: Culicidae) along the Mediterranean and Aegean coasts of Turkey. J Vector Ecol.

[28] Yavasoglu SI, Yilmaz C, Ulger C, Simsek FM (2016). Molecular identification and genetic structure of Aedes phoeniciae (Diptera: culicidae) in Northern Cyprus and Turkey. Biochem Syst Ecol.

[29] Öztürk M, Akiner MM (2023). Mitochondrial cytochrome oxidase I variation in Asian tiger mosquito (*Aedes albopictus*): determination of the different and multiple introduction situations in Turkiye. Acta Zool Academiae Scientiarum Hung.

[30] Akiner MM, Öztürk M (2023). Molecular phylogenetics of *Aedes aegypti* (L., 1762) (Diptera: Culicidae) in Eastern Black Sea area of Turkey and possible relations with the caucasian invasion. Turkish J Zool.

[31] Akıner MM, Öztürk M, Beriş FŞ, Karacaoğlu Ç, Şimşek FM, Akgeyik AU (2022). Distribution and molecular differentiation of Culex pipiens complex species in the Middle and Eastern Black Sea regions of Turkey. Turkish J Zool.

[32] Zawani MKN, Abu HA, Sazaly AB, Zary SY, Darlina MN (2014). Population genetic structure of Aedes albopictus in Penang, Malaysia. Genet Mol Res.

[33] Zoure AA, No G, Sombi A, Somda Z, Badolo A, Francis F (2020). Genetic analysis and population structure of the Anopheles gambiae complex from different ecological zones of Burkina Faso. Infect Genet Evol.

[34] Ergunay K, Gunay F, Kasap E, Oter O, Gargari K, Karaoglu S, Ozkul A (2014). Serological, molecular and entomological surveillance demonstrates widespread circulation of West Nile virus in Turkey. PLoS Negl Trop Dis.

[35] Ergünay K, Litzba N, Brinkmann A, Günay F, Sarıkaya Y, Kar S, Linton YM (2017). Co-circulation of West Nile virus and distinct insect-specific flaviviruses in Turkey. Parasites Vectors.

[36] Hacioglu S, Dincer E, Isler CT, Karapinar Z, Ataseven VS, Ozkul A, Ergunay K (2017). A snapshot avian surveillance reveals West Nile virus and evidence of wild birds participating in Toscana virus circulation. Vector-Borne Zoonotic Dis.

[37] Akıner MM, Öztürk M, Başer AB, Günay F, Hacıoğlu S, Brinkmann A, Ergünay K (2019). Arboviral screening of invasive Aedes species in northeastern Turkey: West Nile virus circulation and detection of insect-only viruses. PLoS Negl Trop Dis.

[38] Longbottom J, Browne AJ, Pigott DM, Sinka ME, Golding N, Hay SI, Moyes CL, Shearer FM (2017). Mapping the spatial distribution of the Japanese encephalitis vector, Culex Tritaeniorhynchus Giles, 1901 (Diptera: Culicidae) within areas of Japanese encephalitis risk. Parasit Vectors.

[39] Xie GL, Ma XR, Liu QY, Meng FX, Li C, Wang J (2021). Genetic structure of Culex Tritaeniorhynchus (Diptera: Culicidae) based on COI DNA barcodes. Mitochondrial DNA Part B Resour.

[40] Li S, Jiang F, Lu H, Kang X, Wang Y, Zou Z, Wen D, Zheng A, Liu C, Liu Q (2020). Mosquito diversity and population genetic structure of six mosquito species from Hainan Island. Front Genet.

[41] Verdonschot PFM, Besse-Lototskaya AA (2014). Flight distance of mosquitoes (Culicidae): a metadata analysis to support the management of barrier zones around rewetted and newly constructed wetlands. Limnologica.

[42] Dusadeepong R, Maquart PO, Hide M, Boyer S (2023). Phylogeny and spatial distribution of Japanese encephalitis virus vector species in Cambodia. Med Vet Entomol.

[43] Napp S, Petrić D, Busquets N (2018). West Nile virus and other mosquito-borne viruses present in Eastern Europe. Pathogens Global Health.

[44] Tavares ES, Baker AJ (2008). Single mitochondrial gene barcodes reliably identify sister-species in diverse clades of birds. BMC Evol Biol.

[45] Lin X, Stur E, Ekrem T (2015). Exploring genetic divergence in a species-rich insect genus using 2790 DNA barcodes. PLoS ONE.

[46] Behura SK (2006). Molecular marker systems in insects: current trends and future avenues. Mol Ecol.

[47] Dong Z, Wang Y, Li C, Li L, Men X (2021). Mitochondrial DNA as a molecular marker in insect ecology: current status and future prospects. Ann Entomol Soc Am.

[48] Schaffner E, Angel G, Geoffro B, Hervy JP, Rhaiem A, Brunhes J (2001) The mosquitoes of Europe. Institute De Recherche pour le Développement (IRD), Montpellier, France. CD-Rom

[49] Folmer O, Black M, Hoeh W, Lutz R, Vrijenhoek R (1994). DNA primers for amplification of mitochondrial cytochrome c oxidase subunit I from diverse metazoan invertebrates. Mol Mar Biol Biotechnol.

[50] Birungi J, Munstermann LE (2002). Genetic structure of Aedes albopictus (Diptera: Culicidae) populations based on mitochondrial ND5 sequences: evidence for an independent invasion into Brazil and United States. Ann Entomol Soc Am.

[51] Hall TA (1999). BioEdit: a user friendly biological sequence alignment editor and analysis program for Windows 95/98/NT. Oxf Univ Press.

[52] Kumar S, Stecher G, Tamura K (2016). MEGA7: molecular evolutionary genetics analysis version 7.0 for bigger datasets. Mol Biol Evol.

[53] Librado P, Rozas J (2009). DnaSP v5: a software for comprehensive analysis of DNA polymorphism data. Bioinformatics.

[54] Excoffier L, Lischer HE (2010). Arlequin suite ver 3.5: a new series of programs to perform population genetics analyses under Linux and Windows. Mol Ecol Resour.

[55] Bandelt HJ, Forster P, Röhl A (1999). Median-joining networks for inferring intraspecific phylogenies. Mol Biol Evol.

[56] Posada D, Crandall KA (1998). MODELTEST: testing the model of DNA substitution. Bioinf (Oxford England).

[57] Provan J, Wattier RA, Maggs CA (2005). Phylogeographic analysis of the red seaweed Palmaria palmata reveals a pleistocene marine glacial refugium in the English Channel. Mol Ecol.

[58] Kumar NP, Rajavel AR, Natarajan R, Jambulingam P (2007). DNA barcodes can distinguish species 1152 of Indian mosquitoes (Diptera: Culicidae). J Med Entomol.

[59] Ashfaq M, Hebert PN, Mirza JH, Khan AM, Zafar Y, Mirza MS (2014). Analyzing mosquito (Diptera: Culicidae) diversity in Pakistan by DNA barcoding. PLoS ONE.

[60] Rajavel AR, Pradeep Kumar N, Natarajan R, Vanamail P, Rathinakumar A, Jambulingam P (2015). Morphological and molecular characterization of the ecological, biological and behavioural variants of the JE vector *Culex Tritaeniorhynchus*: an assessment of its taxonomic status. J Vector Borne Dis.

[61] Maekawa Y, Ogawa K, Komagata O, Tsuda Y, Sawabe K (2016). DNA barcoding for molecular 1266 identification of Japanese mosquitoes. Med Entomol Zool.

[62] Erlanger TE, Weiss S, Keiser J, Utzinger J, Wiedenmayer K (2009). Past, Present, and future of Japanese encephalitis. Emerg Infect Dis.

[63] Karthika P, Vadivalagan C, Thirumurugan D, Kumar RR, Murugan K, Canale A (2018). DNA barcoding of five Japanese encephalitis mosquito vectors (Culex Fuscocephala, Culex Gelidus, Culex Tritaeniorhynchus, Culex pseudovishnui and Culex vishnui). Acta Trop.

[64] Hebert P, Ratnasingham S, de Waard (2003). Barcoding animal life: cytochrome c oxidase subunit 1 divergences among closely related species. Proc R Soc B Biol Sci.

[65] Airi M, Sagandeep K (2015). Confirmation of *Culex (Culex) tritaeniorhynchus summorosus* (Diptera: Culicidae) as a separate species. J Vector Borne Dis.

[66] Jeffries IL, Luciano M, Tantely PK, Marcus SC, Blagrove I, Ioanna L, Orsborne J, Al-Amin HM, Mohammed AR, Alam MS, Girod R, Afrane YA, Bino S, Robert V, Boyer S, Baylis M, Velo E, Hughes GL, Walker T (2022) Genetic and microbial diversity of the invasive mosquito vector species Culex. 10.1101/2022.02.10.479990. tritaeniorhynchus across its extensive inter-continental geographic range 10.12688/wellcomeopenres.20761.1 PMC11128058 38800519

[67] Tajima F (1983). Evolutionary relationship of DNA sequences infinite populations. Genetics.

[68] Tajima F (1996). The amount of DNA polymorphism maintained in a Finite Population when the Neutral Mutation Rate varies among sites. Genetics.

[69] Ramos-Onsins SE, Rozas J (2002). Statistical properties of new neutrality tests against population growth. Mol Biol Evol.

[70] Gugushvili G (2002). Mosquitoes (Diptera: Culicidae) of Georgia. Proc Inst Zool Tbilisi.

[71] Samanidou A, Harbach RE (2003). Culex (Culex) Tritaniorhynchus Giles, a newly discovered potential vector of arboviruses in Greece. J Eur Mosq Control Assoc.

[72] Alves J, Pina ADe, Diallo M, Dia I (2014). First report of Culex (Culex) Tritaeniorhynchus Giles, 1901 (Diptera: Culicidae) in the Cape Verde Islands. Zool Caboverdiana.

[73] Lessard BD, Kurucz N, Rodriguez J, Carter J, Hardy CM (2021). Detection of the Japanese encephalitis vector mosquito Culex tritaeniorhynchus in Australia using molecular diagnostics and morphology. Parasit Vectors.

[74] Verdonschot PFM, Besse-Lototskaya AA (2014). Flight distance of mosquitoes (Culicidae): a metadata analysis to support the management of barrier zones around rewetted and newly constructed wetlands. Limnologica.

[75] Min J-GG, Xue M (1996). Progress in studies on the overwintering of the mosquito Culex tritaeniorhynchus. Southeast Asian J Trop Med Public Health.

[76] Danielovi V, Adhami J (1960). Mosquitoes of Albania and their medical importance. Ceskoslov Parasitol.

[77] Patsoula E, Beleri S, Vakali A, Pervanidou D, Tegos N, Nearchou A (2017) Records of Aedes albopictus (Skuse, 1894) (Diptera; Culicidae) and Culex tritaeniorhynchus (Diptera; Culicidae) Expansion in Areas in Mainland Greece and Islands. Vector-Borne Zoonotic Dis 17(3):217–223. 10.1089/vbz.2016.1974 10.1089/vbz.2016.1974 28075232

[78] Engler O, Savini G, Papa A, Figuerola J, Groschup MH, Kampen H (2013). European surveillance for West Nile virus in mosquito populations. Int J Environ Res Public Health.

[79] Calzolari M (2016). Mosquito-borne diseases in Europe: an emerging public health threat. Rep Parasitol Volume.

